# Biosorption Characteristics of Hg(II) from Aqueous Solution by the Biopolymer from Waste Activated Sludge

**DOI:** 10.3390/ijerph17051488

**Published:** 2020-02-26

**Authors:** Jiao Zhang, Pan Wang, Zhiqiang Zhang, Pengyu Xiang, Siqing Xia

**Affiliations:** 1 School of Civil and Transportation Engineering, Shanghai Urban Construction Vocational College, Shanghai 200432, China; 2 Key Laboratory of Yangtze River Water Environment, Ministry of Education, State Key Laboratory of Pollution Control and Resource Reuse, College of Environmental Science and Engineering, Tongji University, Shanghai 200092, China; 3 Shanghai Jianke Environmental Consulting Co., Ltd., Shanghai 200032, China; 4 Shanghai Institute of Pollution Control and Ecological Security, Shanghai 200092, China; 5 Zhejiang Weiming Environment Protection Co., Ltd., Wenzhou 325000, China

**Keywords:** biopolymer, biosorption, divalent mercury ions (Hg(II)), waste activated sludge

## Abstract

The divalent mercury ion (Hg(II)) is one of the most hazardous toxic heavy-metal ions, and an important industrial material as well. It is essential to remove and recover Hg(II) from wastewater before it is released into the environment. In this study, the biosorption characteristics of Hg(II) from aqueous solution by the biopolymer from waste activated sludge (WAS) are investigated. The major components of the biopolymer consisted of proteins, carbohydrates, and nucleic acids. The adsorption kinetics fit for the pseudo-second-order kinetic model, and the adsorption isotherms were well described by Langmuir equation. The adsorption capacity of the biopolymer increased along with rising temperature, and the maximal adsorption capacity was up to 477.0 mg Hg(II)/g biopolymer at 308 K. The infrared spectroscopy analyses showed that the complexation of Hg(II) by the biopolymer was achieved by the functional groups in the biopolymer, including hydroxyl (–OH), amino (–NH_2_), and carboxylic (–COOH). From the surface morphology, the special reticulate structure enabled the biopolymer to easily capture the metal ions. From the elemental components analyses, a part of Hg(II) ions was removed due to ion exchange with the Na^+^, K^+^, and Ca^2+^, in the biopolymer. Both complexation and ion exchange played key roles in the adsorption of Hg(II) by the biopolymer. These results are of major significance for removal and recovery of Hg(II) from wastewater.

## 1. Introduction

The divalent mercury ion (Hg(II)) is considered one of the most hazardous toxic heavy-metal ions [1]. In aquatic environments, Hg(II) can be microbiologically transformed into methylmercury [2,3]. Methylmercury has very high tendency for binding to proteins, which makes it more prone to bio-magnification [4]. Excessive exposure to Hg(II) can damage the central nervous system and immune system as well as some vital organs like the brain, heart, kidneys, and lungs [3]. The presence of considerably high concentrations of Hg(II) exceeding the common criteria by several times or more has been commonly found in waters from different sources [5]. Hg(II) is an important industrial material, which enters into the water environment mainly through effluents from industrial processes such as chloralkali, electrical equipment, paint and wood pulping, waste incinerators, and mining [6]. Therefore, it is essential to remove and recover Hg(II) from wastewaters or industrial effluents before it is released into the environment.

Biosorbents are believed to be an efficient functional material to remove and recover heavy metal ions from wastewater due to their good efficacy, nontoxicity, and biodegradability [7,8]. Various biomass could become sources for biosorbents, including waste activated sludge (WAS), fungi, bacteria, marine algae, plants, etc. [9,10]. Among the sources, WAS has drawn particular attention because of its excellent metal-capturing performance, cost free nature, and abundant availability [11]. The biopolymer from WAS has been reported to be applied in the removal of heavy metals from industrial wastes [12]. Kusvuran et al. further found that dried WAS could be used to adsorb Pb^2+^, Cd^2+^, and Cu^2+^, from aqueous solution [13]. Moreover, both anaerobic WAS and aerobic WAS were reported to adsorb Cu^2+^, Zn^2+^, Mn^2+^, and Fe^3+^ [14].

There have been some reports about WAS being used to adsorb Hg(II) from wastewater. Sulaymon et al. investigated the competitive adsorption of Pb^2+^, Hg^2+^, Cr^3+^, and As^5+^, from wastewater onto dry WAS, and reported that the adsorption capacity of dry WAS was observed in the order of Pb^2+^ > Hg^2+^ > Cr^3+^ > As^5+^ [15]. Sheng et al. studied the interactions between the extracellular polymeric substances (EPS) and Hg(II) by employing a combination of the zeta potential measurement and the three-dimensional excitation emission matrix (3D-EEM) fluorescence spectroscopy with parallel factor (PARAFAC) analysis [16]. The results revealed that Hg(II) would complex with the functional groups of EPS (e.g., carboxyl), resulting in a change in the EPS conformation and the formation of a stable nonfluorescence complex between EPS and Hg(II). 

Furthermore, the biopolymer extracted from WAS was found to be a matrix of various macromolecules, including proteins, carbohydrates, and nucleic acids [17]. It has been verified that the biopolymer plays a key role in the removal of heavy metals in the activated sludge system for wastewater treatment [18,19]. It was also reported that the biopolymer could be extracted from WAS to serve as a biosorbent for adsorbing heavy metals [20,21]. Since WAS is a byproduct of wastewater biological treatment, the biopolymer as a biosorbent for heavy metals owns ample sources. To our knowledge, there is no report about the adsorption of Hg(II) by the biopolymer up to present. Therefore, it is of major significance to understand the adsorption of Hg(II) by the biopolymer from WAS.

In this study, the biosorption characteristics of Hg(II) from aqueous solution by the biopolymer from WAS were investigated, including adsorption kinetics, isotherms, and thermodynamics. Then, the biosorption mechanisms of Hg(II) by the biopolymer were analyzed via various methods, including infrared spectroscopy, surface morphology, and elemental components analyses.

## 2. Materials and Methods

### 2.1. Biopolymer and Reagents

The WAS samples were collected from the secondary settling tank back-flow sludge from a full-scale municipal wastewater treatment plant in Shanghai, China. The main parameters of the sludge after gravity concentration were as follows: pH 6.8–7.5, suspended solids (SS) 9.0 ± 1.0 g/L, and the ratio of volatile suspended solids to suspended solids (VSS/SS) 65 ± 8%. The extraction of the biopolymer was carried out with an ultrasound followed by centrifugation. The ultrasound reactor was equipped with a transducer (20 kHz, diameter of 13 mm). During ultrasonic treatment (power density 2.7 kW/L, pulse 4 s), the tip of the transducer was immersed at about 10 mm deep into 100 mL sludge samples to be processed for 2 min, and the temperature was maintained at about 298 K. Then, the treated sludge samples were centrifuged twice at 10,800 × g and 277.15 K for 10 min every time. The supernatant was the raw biopolymer. To purify the biopolymer, three volumes of cold acetone were added to the rude liquid biopolymer. After staying in fridge (277.15 K) for about 12 h, the mixture was centrifuged at 277.15 K and 15,777 × g for 10 min. The obtained precipitate was redissolved in distilled water to the original volume, and then the above operations were repeated two times more. The precipitate obtained at the third time was dialyzed in distilled water overnight at 277.15 K, and finally was lyophilized to obtain the purified biopolymer [21]. The biopolymer consisted of proteins (54.76%, w/w), polysaccharides (30.43%, w/w), and nucleic acids (14.81%, w/w).

All of the used chemical agents, which were of analytical grades, were obtained from Runjie Chemistry Reagents Co. Ltd. (Shanghai, China). The stock solution of Hg(II) with an initial concentration of 1,000 mg/L was prepared by dissolving analytical reagent (AR) grade of HgSO_4_ into distilled water.

### 2.2. Adsorption Kinetics Analyses

The adsorption kinetic experiments were conducted in an isothermal shaker (HAQ-F160, Donglian Electronic Technology Development, Harbin, China) under the following conditions: initial Hg(II) concentrations 10, 20, and 40 mg/L; the mass ratio of the biopolymer to Hg(II) 2.2/1; initial pH 5.0; solution volume 50 mL; reaction temperature 298 K; and shaking speed 150 r/min. During the adsorption process, the solution was sampled with a syringe at set times. The supernatant of the sample was separated from the precipitates by centrifugation at 10,800 × g for 10 min and then filtrated using a 0.22 μm cellulose nitrate membrane filter. The Hg(II) concentration in the supernatant was measured by inductively coupled plasma atomic emission spectrometry (ICP-AES, Agilent 720ES, Santa Clara, CA, USA). The adsorption capacity of the biopolymer for Hg(II) was calculated using Equation (1).

(1) $$q_{e}=\frac{\left(C_{0}-C_{e}\right)V}{W}$$

where *C*_0_ (mg/L) and *C_e_* (mg/L) were Hg(II) concentrations in solution at time 0 and equilibrium state, respectively; *q_e_* (mg/g) was the adsorption capacity of the biopolymer at equilibrium status; *V* (L) was the volume of the adsorbate used; and *W* (g) was the mass of the biopolymer.

### 2.3. Adsorption Isotherms and Thermodynamics Analyses

Batch experiments were conducted to investigate the thermodynamic properties of Hg(II) by the biopolymer in an isothermal shaker (HAQ-F160, Donglian Electronic Technology Development, Harbin, China) under the following conditions: initial Hg(II) concentrations 5, 10, 20, 30, and 40 mg/L; the mass ratio of the biopolymer to Hg(II) 2.2/1; initial pH 5.0; solution volume 50 ml; reaction temperature 298 K; and shaking speed 150 r/min. After 120 min of reaction time, the Hg(II) concentration in the sampled supernatant was analyzed as stated before.

Based on the results of adsorption isotherms of Hg(II) by the biopolymer, the adsorption thermodynamics were analyzed according to Equations (2)–(4) [22].

(2) $$\Delta G^{0}=-RT\ln K_{L}\;$$

(3) $$\ln K_{L}=\frac{\Delta S^{0}}{R}-\frac{\Delta H^{0}}{RT}$$

(4) $$\Delta G^{0}=\Delta H^{0}-T\Delta S^{0}\;$$

where *R* (= 8.314 J/mol·K) is the ideal gas constant; *T* (K) is the absolute temperature; *K_L_* is the equilibrium constant of adsorption; and Δ*G*^0^, Δ*H*^0^, and Δ*S*^0^ are Gibbs free change, enthalpy change, and entropy change, respectively.

### 2.4. Physicochemical Analyses

Chemical analyses of the biopolymer were conducted to identify the components of the biopolymer. The biopolymer was mainly composed of proteins, polysaccharides, and nucleic acids. The proteins content was determined by the Bradford method using bovine serum albumin (BSA) as the standard [23]. The polysaccharides content was measured by the phenol-sulfuric acid method with glucose as the standard solution [24]. The nucleic acids content was measured by the diphenylamine colorimetric method using calf thymus deoxyribonucleic acid as the standard [25].

The infrared spectra were obtained as KBr pellets (1 wt%) at room temperature, and the equipment used was Fourier transform infrared spectroscopy (FTIR) spectrophotometer Nicolet 5700 in the wave number range of 4000–500 cm^−1^.

The elemental components were analyzed by a scanning electron microscopy (SEM)/energy-dispersive X-ray (EDX), and images were obtained by a Philips XL 30 ESEM-FEG (FEI, Eindhoven, Netherlands) with an EDX spectroscopy (EDAX, Mahwah, NJ, USA). The main parameters of the equipment were set as follows: accelerating voltage 15 kV, activity time 50 s, and counts per second (CPS) 1500.

## 3. Results and Discussion

### 3.1. Adsorption Kinetics of Hg(II) by the Biopolymer

The adsorption kinetics experiments were conducted at three initial Hg(II) concentrations (10, 20, and 40 mg/L). Both pseudo-first-order model and pseudo-second-order model were used to investigate the adsorption kinetics of Hg(II) by the biopolymer [26]. From Table 1, pseudo-second-order model fitted the experimental data better than pseudo-first-order model under any initial Hg(II) concentration. It seems that the rate-limiting step of the adsorption might be chemical sorption [27]. The adsorption process could be divided into three stages: the fast sorption, the slow sorption, and the equilibrium. The fast sorption stage was determined at the very beginning of adsorption (0–10 min), when the sorption rate was extremely high. When extending the adsorption time from 10 to 60 min, the slow sorption stage showed a decreasing sorption rate. The amount of Hg(II) adsorbed at equilibrium was obtained at the time of 120 min and has been maintained ever since (not shown), indicating that the adsorption process was at equilibrium stage. The *q_e_* was raised from 441.4 to 497.4 mg Hg(II)/g biopolymer with an increase in the concentration of Hg(II) from 10 to 40 mg/L, suggesting that increasing Hg(II) concentration was helpful to improve the adsorption capacity of the biopolymer.

### 3.2. Adsorption Isotherms and Thermodynamics of Hg(II) by the Biopolymer

The adsorption isotherms at three temperatures were investigated. The adsorption of Hg(II) by the biopolymer was described using both Langmuir and Freundlich isotherm models (Table 2). The values of R^2^ of Langmuir model at 288, 298, and 308 K were found to be 0.9916, 0.9748, and 0.9832, respectively, indicating that the biosorption of Hg(II) by the biopolymer fitted well the Langmuir model. In other words, the adsorption of Hg(II) by the biopolymer took place at the functional groups/binding sites on the surface of the biopolymer, which is regarded as monolayer biosorption [28]. The binding sites might be heterogeneous due to presenting *n* > 1 in Freundlich model [29]. In order to identify the best-fit isotherm model, linear Chi-square values (*χ*^2^) were examined by using the models of Equation (5) [30].

(5) $$\chi^{2}=\sum \frac{{\left(q_{e}-q_{cal}\right)}^{2}}{q_{cal}}\;\;$$

where *q_e_* was stated as in Equation (1) and *q_cal_* was (mg/g) was the adsorption capacity of the biopolymer obtained from isotherms.

When data obtained using a model are similar to the experimental data, *χ*^2^ is close to zero. High *χ*^2^ represents a high bias between the experiment and model [30]. The Chi-square values of the Langmuir model (38.11, 28.32, and 0.73) were found to be lower than those of the Freundlich model (38.77, 33.96, and 1.50). Therefore, the adsorption isotherms of Hg(II) by the biopolymer were clearly described by the Langmuir model.

The adsorption thermodynamics at the three temperatures were investigated. The results are shown in Table 3. The Δ*G*^0^ values lower than 20 kJ/mol indicate that the adsorption process of Hg(II) by the biopolymer was spontaneous, and both physical adsorption and chemical adsorption took place. The Δ*H*^0^ value above 0 denotes that the adsorption process was endothermic. The Δ*S*^0^ value above 0 means that the adsorption process improved the disorder at the solid/solution interface, with some structural changes in the adsorbate and the adsorbent and an affinity of the adsorbent [22].

The reported biosorbents for Hg(II) removal in the literature include rice straw, dry activated sludge, and moss biomass. As shown in Table 4, great differences are found between the maximum Hg(II) adsorption capacities of the reported biosorbents and the biopolymer in this study. The maximum Hg(II) adsorption capacity obtained in this study is markedly higher than the reported values, demonstrating the potential applicability of the biopolymer as an excellent adsorbent.

### 3.3. Adsorption Mechanisms of Hg(II) by the Biopolymer

#### 3.3.1. Functional Groups Analyses

**Figure 1:**
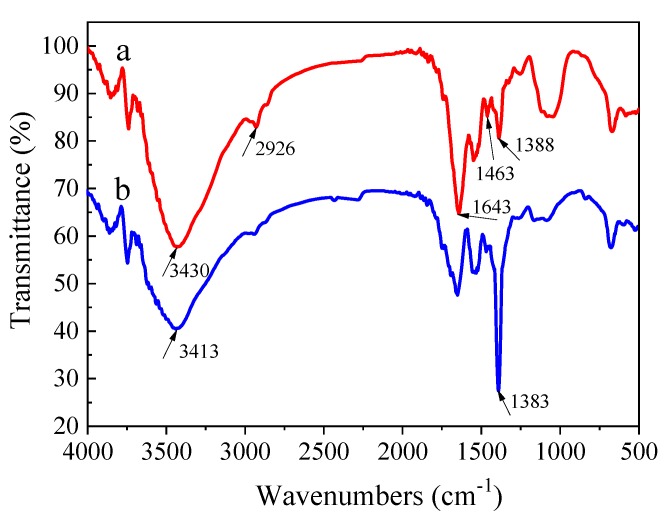
Fourier transform infrared spectroscopy (FTIR) spectra of the biopolymer before (**a**) and after (**b**) adsorbing Hg(II).

The FTIR spectroscopy method was used to better understand the nature of the functional groups responsible for the adsorption process. Figure 1 shows the FTIR spectra of the biopolymer before and after adsorbing Hg(II). Hydroxyl (–OH), amino (–NH_2_), carbonyl (–C=O), and carboxylic (–COOH) groups were found in the biopolymer. The peak around 3430 cm^−1^ was dominated by –OH and –NH_2_ stretching. The peak at 2926 cm^−1^ is characterized as the typical one of sugar, which was mainly caused by C–H stretching vibrations. The peaks at 1463 cm^−1^ and 1388 cm^−1^ suggests the existence of –C=O and carboxylic –COOH groups, respectively.

The FTIR spectra changed a lot after the biopolymer adsorbing Hg(II). The broad intense peak around 3430 cm^−1^ weakened and shifted to 3413 cm^−1^, indicating that –OH and –NH_2_ played a role in the adsorption of Hg(II) by the biopolymer. The peak of 1388 cm^−1^ obviously increased and shifted to 1383 cm^−1^, and the peak at 1643 cm^−1^ significantly weakened. This appearance resulted from the –C=O decreasing and the corresponding –C=O increasing due to the H ions in –COOH replaced by Hg(II). The results of the FTIR spectra demonstrated that the functional groups responsible for the binding of Hg(II) by the biopolymer might chiefly consiste of –OH, –NH_2_, and –COOH.

#### 3.3.2. Surface Morphology Analyses

**Figure 2:**
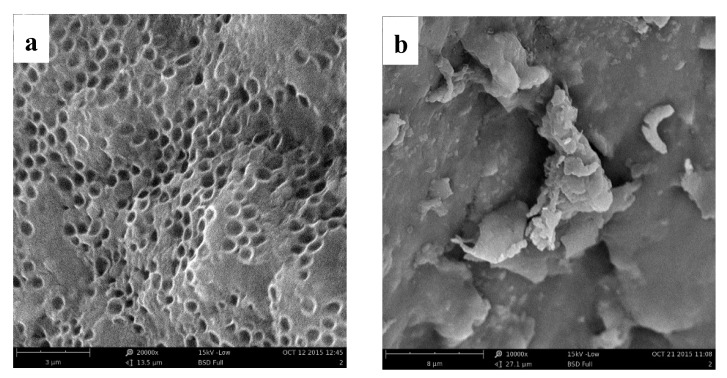
Surface morphology of the biopolymer before (**a**) and after (**b**) adsorbing Hg(II).

The surface morphology images of the biopolymer before and after adsorb Hg(II) are shown in Figure 2. With a special reticulate structure, the biopolymer easily captured the metal ions during the adsorption process, which became precipitates that were separated from the solutions. After adsorbing Hg(II), the precipitates were found to be compact and the meshes in the biopolymer disappeared. The results denoted that the biopolymer owned excellent adsorption performance for Hg(II).

#### 3.3.3. Elemental Components Analyses

The EDX analyses were done to determine the contents of elements in the biopolymer before and after adsorbing Hg(II). The EDX spectra results of the samples are presented in Table 5. Besides C and O, the major elements found in the biopolymer were P, N, Na, Ca, and K. The content of Na, Ca, and K was 1.5%, 1.5%, and 0.8%, respectively. After adsorbing Hg(II), the peaks of Na, Ca, and K disappeared and the peaks of P and N clearly weakened. Contrary to the lack of the above elements, the atomic percentage of Hg(II) increased from 0% to 47.1% greatly. The EDX analyses confirmed that Hg(II) was removed because of metal ions in solution exchanging with the elements of Na, Ca, and K in the biopolymer during the adsorption process.

According to the above adsorption kinetics, isotherms, and thermodynamics analyses, the adsorption of the biopolymer for Hg(II) was physisorption-enhanced by chemisorption. The FTIR, SEM, and the EDX analyses further revealed that complexation and ion exchange between the functional groups and Hg(II) played key roles in the chemisorption.

## 4. Conclusions

The present study examined the biosorption characteristics of Hg(II) by the biopolymer from WAS and further disclosed its adsorption mechanisms. The pseudo-second-order kinetic model better fit the adsorption kinetics. Langmuir equation better described the adsorption isotherms. The maximal adsorption capacity was up to 477.0 mg Hg(II)/g biopolymer at 308 K. The complexation of Hg(II) by the biopolymer was achieved by the functional groups in the biopolymer, including –OH, –NH_2_, and –COOH. The special reticulate structure made the biopolymer easily capture the metal ions. Part of Hg(II) was removed due to ion exchange with the Na^+^, K^+^, and Ca^2+^, in the biopolymer. Over 85% of Hg(II) was recovered from the biopolymer-Hg(II) complex with EDTA at pH 2.5. Both complexation and ion exchange played key roles in the adsorption of Hg(II) by the biopolymer.

## Figures and Tables

**Table 1:** Kinetic parameters obtained from pseudo-first-order and pseudo-second-order for the adsorption of divalent mercury (Hg(II)) by the biopolymer for different initial Hg(II) concentrations.

*C* (mg/L)	*q*_e, exp_ (mg/g)	Pseudo-First-Order Model			Pseudo-Second-Order Model		
		*q*_cal_ (mg/g)	*K*_1_ (min^−1^)	R^2^	*q*_cal_ (mg/g)	*K*_2_ (g/(mg·min))	R^2^
10	441.4	225.7	0.06351	0.9042	471.0	0.0005250	0.9719
20	492.3	295.3	0.06433	0.9431	525.0	0.0004200	0.9605
40	497.4	254.7	0.05544	0.9059	519.6	0.0005180	0.9218

**Table 2:** Isotherm parameters obtained from the Langmuir and Freundlich models for the adsorption of Hg(II) by the biopolymer at different temperatures.

Temperature (K)	Langmuir Model			Freundlich Model		
	*q*_m__ax_ (mg/g)	*K*_L_ (L/mg)	R^2^	*K*_F_ (mg/g)(L/mg)^1/*n*^	*n*	R^2^
288	423.6	0.0953	0.9916	79.74	2.507	0.9401
298	452.9	0.1505	0.9748	129.8	3.358	0.9073
308	477.0	0.2217	0.9832	180.5	4.109	0.9689

**Table 3:** Adsorption thermodynamics parameters of Hg(II) by the biopolymer.

Δ*G*^0^ (kJ/mol)			Δ*H*^0^ (kJ/mol)	Δ*S*^0^ (J/mol·K)	*R* ^2^
288 K	298 K	308 K			
−23.61	−25.54	−27.39	30.93	189.4	0.9998

**Table 4:** Comparison of the maximum adsorption capacity of Hg(II) in literature and this study.

Biosorbent	pH	Temperature (K)	*q*_e_	Reference
Rice straw	5.0	298	22.1	[29]
Dry activated sludge	4.0	298	10.8	[15]
Moss (*Drepanocladus revolvens*) biomass	5.5	293	94.4	[28]
EPS from activated sludge	7.4	293	452.8	[31]
Chemical activation of dried sewage sludge (AS)	5.0	298	106.4	[32]
Gum karaya	6.0	298	62.6	[33]
Raw activated sludge	7.0	308	57.8	[34]
Biopolymer	5.0	308	477.0	In this study

**Table 5:** The content of elements in the biopolymer before and after adsorption of Hg(II).

Elements	Atomic Percentage (%) before Adsorption of Hg(II)	Atomic Percentage (%) after Adsorption of Hg(II)
O	38.9	7.4
C	30.0	41.3
N	24.1	4.0
P	3.1	0.1
Na	1.5	0.1
Ca	1.5	0
K	0.8	0
Hg	0	47.1
